# Numerical Simulations of the Low-Velocity Impact Response of Semicylindrical Woven Composite Shells

**DOI:** 10.3390/ma16093442

**Published:** 2023-04-28

**Authors:** Luis M. Ferreira, Carlos A. C. P. Coelho, Paulo N. B. Reis

**Affiliations:** 1 Grupo de Elasticidad y Resistencia de Materiales, Escuela Técnica Superior de Ingeniería, Universidad de Sevilla, Camino Descubrimientos, 41092 Sevilla, Spain; lmarques@us.es; 2 Escuela Politécnica Superior, Universidad de Sevilla, C/Virgen de África, 7, 41011 Sevilla, Spain; 3 Unidade Departamental de Engenharias, Escola Superior de Tecnologia de Abrantes, Instituto Politécnico de Tomar, Rua 17 de Agosto de 1808, 2200-370 Abrantes, Portugal; cccampos@ipt.pt; 4 University of Coimbra, CEMMPRE, ARISE, Department of Mechanical Engineering, 3030-194 Coimbra, Portugal

**Keywords:** low-velocity impact, finite element method (FEM), woven-fabric composites

## Abstract

This paper presents an efficient and reliable approach to study the low-velocity impact response of woven composite shells using 3D finite element models that account for the physical intralaminar and interlaminar progressive damage. The authors’ previous work on the experimental assessment of the effect of thickness on the impact response of semicylindrical composite laminated shells served as the basis for this paper. Therefore, the finite element models were put to the test in comparison to the experimental findings. A good agreement was obtained between the numerical predictions and experimental data for the load and energy histories as well as for the maximum impact load, maximum displacement, and contact time. The use of the mass-scaling technique was successfully implemented, reducing considerably the computing cost of the solutions. The maximum load, maximum displacement, and contact time are negligibly affected by the choice of finite element mesh discretization. However, it has an impact on the initiation and progression of interlaminar damage. Therefore, to accurately compute delamination, its correct definition is of upmost importance. The validation of these finite element models opens the possibility for further numerical studies on of woven composite shells and enables shortening the time and expenses associated with the experimental testing.

## 1. Introduction

Due to its distinctive combination of high strength, low weight, and exceptional fatigue resistance, composite materials have grown in popularity. Nevertheless, low-velocity impacts that may happen during handling, transit, maintenance, and service might harm them. These collisions may result in localized structural damage, which may diminish the composite material’s strength, stiffness, and durability. To ensure the dependability and safety of composite structures, it is crucial to comprehend the behavior of composite materials under low-velocity impacts.

In a low-velocity impact event, composite materials undergo various stages of damage. Firstly, when the impactor contacts with the material, a sudden increase in stress and strains is experienced in a very localized region. Secondly, microcracks start to develop in the material matrix, which then propagate through the material and spread to the adjacent laminas and neighboring interface regions. At this stage, the damage progression causes the separation of the adjacent laminas of the composite material, which is known as delamination. This damage mechanism, alongside with matrix cracking, fiber failure and perforation, can significantly reduce the strength and stiffness of the material. The extent and severity of damage depends on the properties of the composite material, the impactor’s properties, and the impact energy. The development of reliable models capable of predicting these damage stages is of highest importance for the analysis and design of composite structures that are resistant to low-velocity impacts.

In this context, the behavior of composite structures has been extensively studied using finite element (FE) models in a variety of industries, including the marine, automotive, and aerospace sectors [1,2,3,4,5]. The impact response of composite flat plates has been extensively studied using numerical models to examine the impacts of various parameters such as material characteristics, impact energy, and geometry. Yet, there are relatively few numerical studies that analyze the impact dynamics on cylindrical shells, particularly when it comes to composites made of woven fabric. The most relevant numerical studies that were conducted on cylindrical shells include the work developed by Kim et al. [6] in which they observed that the contact force increases with the curvature of shell-shaped composite laminates, while the deflection and contact time decrease. It was also observed that the impactor’s velocity has a greater influence on the contact force than the impactor’s mass, which is similar to the impact response of composite plates [6,7,8]. This is justified by the fact that the kinetic energy of the impactor increases linearly with the increase in mass and quadratically with velocity. The contact force and deflection histories for composite laminated cylindrical shells with convex and concave shapes were analyzed by Choi [9]. The author found the same contact force and central deflection histories for both shapes. Kistler and Wass [10] performed a numerical study on unidirectional (UD) laminated cylindrical shells and identified scaling relationships between impact energy, momentum, mass, and velocity, while Zhao and Cho [11] investigated the impact-induced damage initiation and propagation of UD composite shells and found that the damage propagates differently from composite flat plates. Another study was performed by Kumar et al. [12] to study the impact response and impact-induced damages of cylindrical UD composite laminate shells using a 3D finite element formulation. Recently, Khalili et al. [13] used FE analysis to investigate the impact response of UD composite laminate plates and shells structures under low-velocity impact loads and optimize the procedure for future work. Albayrak et al. [14] conducted an experimental and numerical investigation to study the geometrical effect on low-velocity impact behavior for curved composites with a rubber interlayer. They found that the curved surface geometry affects the absorbed energy and that increasing the width of the laminate while keeping the height constant results in higher impact energy absorption.

Overall, these studies provide valuable insights into the behavior of composite laminate structures under low-velocity impacts and can inform about the design and optimization of such structures for various applications. Nevertheless, none of these numerical studies was dedicated to the development of FE models capable of analyzing the low-velocity impact response of semicylindrical woven fabric composite shells. Therefore, the main goal of this paper is to develop reliable and efficient FE models capable of predicting the impact behavior of these materials. For this purpose, constitutive modes that consider the intralaminar and interlaminar progressive damage were implemented in the explicit finite element approach using ABAQUS/Explicit [15]. The validation of the FE models was carried out using the authors’ previous work on the experimental assessment of the effect of thickness on the multi-impact response of semicylindrical composite laminated shells [16]. To facilitate the understanding, the nomenclature used in this paper is listed in the Nomenclature.

## 2. Material and Experimental Procedure

**Figure 1:**
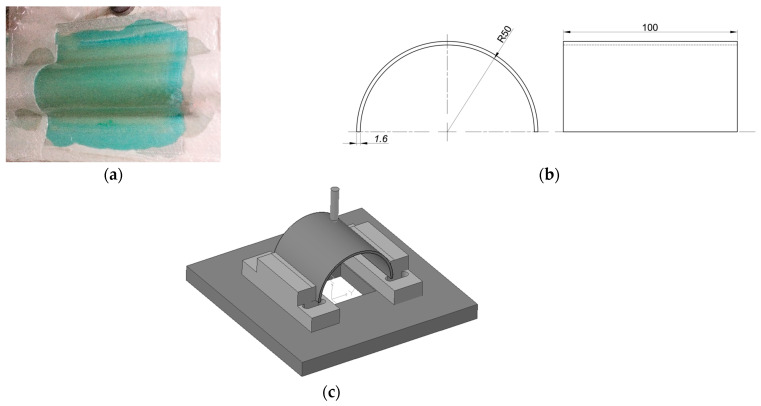
(**a**) Manufacturing process; (**b**) Geometry and dimensions of the specimens (in mm); (**c**) Schematic view of the test conditions.

Composite semicylindrical shells were produced using a matrix based on an AROPOL FS 1962 polyester resin and a MEKP-50 hardener (both supplied by SF Composites, Mauguio, France). A bi-directional E-glass woven fabric (taffeta with 210 g/m^2^) was used as reinforcement, and the composite was produced by hand lay-up with 9 woven fabric layers (corresponding to 1.6 mm of final thickness). In order to ensure a constant fiber volume fraction and uniform thickness, as well as to eliminate any air bubbles, the laminates were placed inside a vacuum bag immediately after impregnation. The manufacturing process culminated with curing at 40 °C for 24 h. More details about the materials and manufacturing process can be found in [16,17,18]. Figure 1 shows the specimens’ dimensions and the schematic view of the test conditions.

Finally, low-velocity impact tests were performed on a drop weight testing machine IMATEK-IM10 (Old Knebworth, UK), which is described in detail in [19]. These tests were carried out according to ASTM D7136 standard, at room temperature, and using an impactor diameter of 10 mm with a mass of 2.826 kg. The energy of 5 J was used to promote visible damage but without full perforation. More details about these tests can be found in [16].

## 3. Damage Models

Two constitutive models were used in this study to simulate the material damage caused by low-velocity impact loads in semicylindrical composite laminate shells: (i) a continuum damage model (CDM) at the lamina level to account for intralaminar damage; and (ii) a surface-based cohesive damage model (S-BCM) at the lamina interface to account for interlaminar damage.

The built-in constitutive model for fabric-reinforced composites available in Abaqus/Explicit [15], developed by Johnson [20] and based on Ladeveze and Ledantec work [21], was used to evaluate the complex damage progression at the intralaminar level. When the user-defined material is named with the string “ABQ PLY FABRIC”, this model can be used as a built-in VUMAT user subroutine [22].

The maximum stress criterion determines the damage initiation, and the fracture energies serve as the basis for the damage evolution model, which regulates the decline in stiffness. In this way, the following damage activation functions, $F_{\alpha }$ and $F_{12}$, are used to compute the elastic domain at any given time,

(1) $$F_{\alpha }=\frac{{\tilde{\sigma }}_{\alpha }}{X_{\alpha }}-r_{\alpha }\leq 0\;\mathrm{with}\;\alpha =1\pm ,2\pm$$

(2) $$F_{12}=\frac{{\tilde{\sigma }}_{12}}{S_{12}}-r_{12}\leq 0$$

where ${\tilde{\sigma }}_{\alpha }$ and ${\tilde{\sigma }}_{12}$ are the effective normal and shear stresses, respectively, $X_{\alpha }$ is the tensile/compressive strength, $S_{12}$ is the shear strength, and $r_{\alpha }$ and $r_{12}$ are the corresponding damage thresholds, which are initially set to 1. Once damage is predicted, the elastic–stress–strain relations are given by,

(3) $$\varepsilon =\left[\begin{array}{ccc} \frac{1}{\left(1-d_{1}\right)E_{1}} & -\frac{\nu_{12}}{E_{1}} & 0\\ -\frac{\nu_{12}}{E_{2}} & \frac{1}{\left(1-d_{2}\right)E_{2}} & 0\\ 0 & 0 & \frac{1}{\left(1-d_{12}\right)2G_{12}} \end{array}\right]\;\sigma$$

where $d_{1}$ and $d_{2}$ are the damage variables associated with fiber fracture along directions 1 and 2, respectively, and $d_{12}$ is the damage variable associated with the matrix microcracking due to shear deformation. These variables are determined with Equations (4) and (5),

(4) $$d_{\alpha }=1-\frac{1}{r_{\alpha }}e^{-A_{\alpha }\left(r_{\alpha }-1\right)}\;\mathrm{with}\;A_{\alpha }=\frac{2g_{0}^{\alpha }L_{e}}{G_{f}^{\alpha }-g_{0}^{1,2}L_{e}}\;\mathrm{and}\;g_{0}^{\alpha }=\frac{X_{\alpha }^{2}}{2E_{\alpha }}\;$$

(5) $$d_{12}=min\left[\alpha_{12}\ln\left(r_{12}\right),\;d_{12}^{max}\right]$$

where $r_{\alpha }$ and $r_{12}$ stand for the damage thresholds for axial and shear loads, respectively, $G_{f}^{\alpha }$ stands for the fracture energies, $g_{0}^{\alpha }$ to the elastic energy density, $L_{e}$ stands for the characteristic length of the element, and $\alpha_{12}$ stands for the shear damage parameter.

The non-linear behavior of the matrix, due to microcracking, dominates the shear damage response at the intralaminar level and includes both stiffness reduction and plasticity. The latter is defined with the following yield and hardening functions; see Equations (6) and (7), respectively.

(6) $$F_{pl}=\left|{\tilde{\sigma }}_{12}\right|-{\tilde{\sigma }}_{0}\left({\bar{\varepsilon }}^{pl}\right)\;\leq 0\;\;$$

(7) $${\tilde{\sigma }}_{0}\left({\bar{\varepsilon }}^{pl}\right)={\tilde{\sigma }}_{y0}+C{\left({\bar{\varepsilon }}^{pl}\right)}^{p}\;$$

where ${\tilde{\sigma }}_{y0}$ and ${\tilde{\sigma }}_{0}$ are the initial effective shear stress and shear yield stress, ${\bar{\varepsilon }}^{pl}$ is the plastic strain due to shear deformation, and C and the superscript p correspond to the coefficient and power term in the hardening function, respectively.

Using the two-step homogenization methodology described by Liu et al. [23], the stiffness properties of the woven fabric composite laminas were estimated from the constituents’ properties of the tested specimens in order to validate the numerical model based on the experimental evidence presented in [16]. The values of the remaining intralaminar material properties were taken from the literature. In this way, the fracture toughness values, the damage evolution parameters, $\alpha_{12}\;\mathrm{and}\;d_{12}^{max}$, and the shear plasticity parameters, ${\tilde{\sigma }}_{y0},\;C\;\mathrm{and}\;p$, were taken from impact studies on E-glass laminates [24,25,26], whereas the strength properties were taken from impact studies on woven E-glass/polyester composite laminates [27,28,29]. The intralaminar material parameters needed to specify the material model in the VUMAT subroutine are shown in Table 1. It is noteworthy to mention that a preliminary parametric study was performed using a coarser FE mesh to assess how a reasonable variation of the shear plasticity parameters could impact the results. It was found that they have a negligible effect on the numerical solutions. Regarding the strength properties, the data found in the literature vary substantially, depending on the manufacturing process, type e-glass fiber, volume fraction, etc. Taking this fact into consideration, the values employed are based on averaged values and again, a preliminary parametric study was performed with a coarser FE mesh to assess which values best fit the experimental evidence. Finally, to account for the interlaminar damage, the bond between the laminas of the composite laminate was modeled using cohesive surfaces. This approach is primarily intended for negligible small interface thicknesses and offers very similar capabilities to cohesive elements. The cohesive behavior is defined as a surface interaction property, and identically to the cohesive elements, it is governed by a traction–separation constitutive model. The properties employed in the surfaced-based cohesive model shown in Table 2 were extracted from the literature [30,31,32,33,34,35,36,37,38].

The cohesive stiffness in this study is set at 10^6^ N/mm^3^, as suggested by Camanho et al. [30]. In addition, it is considered that its value is the same for all directions, that is, $k_{n}=k_{s}=k_{t}$, as used in [31,32,33] with satisfactory results. It is noteworthy to mention that considering high values for the cohesive stiffness potentially results in convergence problems. On the other hand, the use of low values may affect the global stiffness and thus compromise the validation of the FE model [32]. A value of η=1.45 was considered for the interaction parameter in the definition of the cohesive model [34,35,36,37,38]. Identically to the intralaminar properties, there is a wide range in the data for the interlaminar strength parameters and fracture toughness. Given this information, the values used are averages, and preliminary parametric analysis using a coarser FE mesh was completed to determine which values the best-matched experimental data.

## 4. Finite Element Model

**Figure 2:**
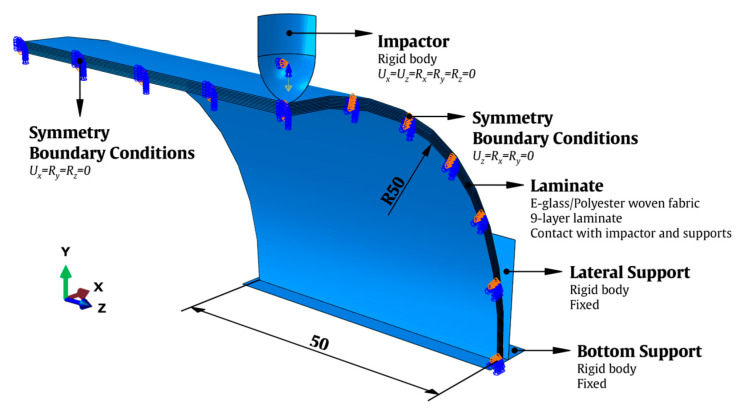
FE model with geometric parameters and boundary conditions.

The FE model was created using the ABAQUS/Explicit FE code [15] taking into account the dimensions of the nine-layer laminate specimens evaluated in [16]. The tested specimens had a semicircular internal radius of 50 mm, a length of 100 mm and an average thickness of 1.6 mm (Figure 2).

To replicate the experiments, two fixed rigid body supports (a lateral and a bottom support) were included in the FE model. Only one-fourth of the semicylindrical composite laminate was generated, taking use of the geometric symmetry of the model to reduce the computing cost of the numerical simulations. The *yz*-plane face $\left(U_{x}=R_{y}=R_{z}=0\right)$ and one of the *xy*-plane faces $\left(U_{z}=R_{x}=R_{y}=0\right)$ were therefore added to the symmetry boundary conditions. The impactor was modeled with a lumped mass fixed on a reference point at its center of mass equivalent to the experiments and with a hemispherical head with a diameter of 10 mm. Only the displacements in the *y*-direction were permitted $\left(U_{x}=U_{z}=0\right)$, and all the rotations of the impactor were constrained $\left(R_{x,y,z}=0\right)$.

Each lamina was discretized with SC8R continuum shell elements (eight-node hexahedron) with reduced integration and stiffness hourglass formulation. The orientations of the materials along the semicircular cross-section were taken into account when defining the local coordinate system of the laminas. R3D4 discrete rigid elements were used to model the impactor. The lamina was modeled with cohesive surfaces; thus, no element specification was required.

The surface-to-surface contacts between the composite laminate, the metal impactor, and the metal supports were simulated using the penalty enforcement contact method from Abaqus/Explicit [15]. In the interface of the composite laminas, which experience friction after being entirely delaminated, this contact formulation was also defined. The friction coefficient values, μ, used in this work for fully damaged interfaces and metal–composite contacts were taken from [39,40]. A value of μ=0.3 was specified for the contact between the metal hemispherical head of the impactor and the upper surface of the composite laminate, and a value of μ=0.7 was specified for the contact between the metal surfaces of the supports and the composite laminate surfaces. For the interfaces, a value of μ=0.5 was considered.

## 5. Numerical Results

Several numerical simulations were performed to determine the influence of the FE mesh discretization and mass scaling on the efficiency and reliability of the FE model. To be able to analyze how these parameters affect the numerical predictions, the most relevant load and energy histories are presented, as well as the maximum force, displacement, and contact time.

The use of cohesive surfaces implies that its elements’ characteristic length matches the characteristic length of the continuum shell elements defined for the laminas. Given that the interlaminar damage surface-based cohesive damage model implementation yields mesh-dependent results, the FE mesh discretization of the laminas needs to be defined based on the characteristic length of the cohesive surface elements. In other words, the FE mesh size of the interface defines the size of the FE mesh employed in the whole model.

To find a balance between the computational cost of the solution and the accurate computation of the fracture toughness in the interlaminar damage model, which results in delamination, a parametric study was conducted to optimize the characteristic length of the elements of the FE mesh. Based on the work of Hilleborg et al. [41], Turon et al. [31] purposed using Equations (8) and (9) to calculate the characteristic length of the element in the direction of the crack propagation for fracture modes I, II, and III in orthotropic composite materials,

(8) $$l_{e,I}=\frac{ME_{3}G_{Ic}}{N_{e}{\left(\tau_{n}^{0}\right)}^{2}}$$

(9) $$l_{e,II}=l_{e,III}=\frac{MG_{13}G_{IIc}}{N_{e}{\left(\tau_{s}^{0}\right)}^{2}}$$

where M is a parameter that depends on the cohesive zone model, and $N_{e}$ is the number of elements in the cohesive zone. The lowest value derived from the equations is $l_{e,II}=0.31$ mm, with the assumptions that M=1, as suggested in [23,33,35], $N_{e}=5$ to properly establish the cohesive zone [42,43,44], and the baseline properties of the laminas and of the cohesive zone. Consequently, the baseline FE mesh was generated with $l_{e}=0.3\;\mathrm{mm}$, which corresponds to an aspect ratio of 1.6. Notice that this value is in good agreement with that used by Lopes et al. in [35].

**Figure 3:**
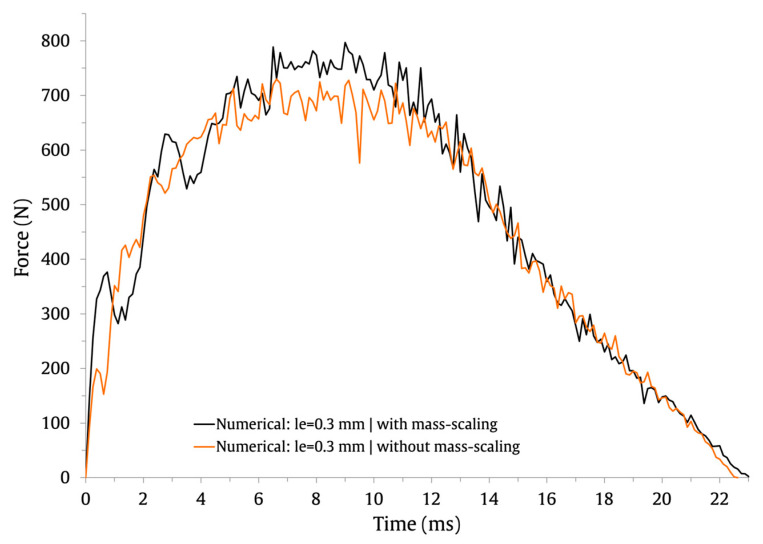
Effect of mass scaling on the force–time impact curves.

**Figure 4:**
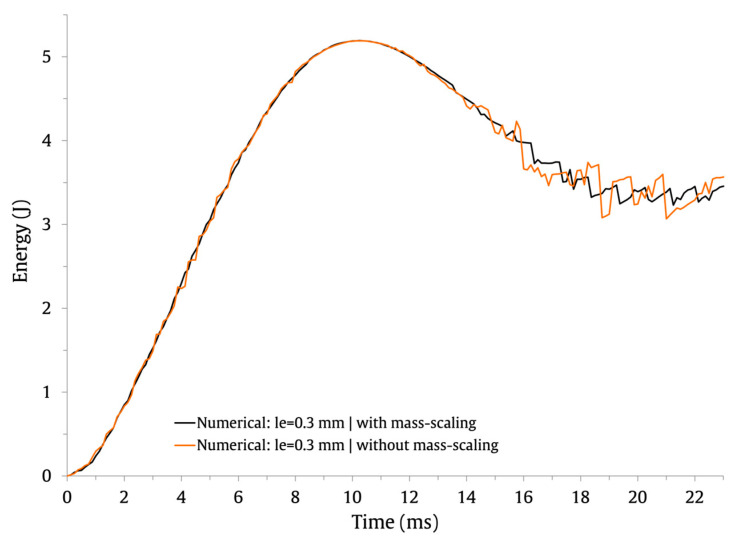
Effect of mass scaling on the energy–time impact curves.

**Figure 5:**
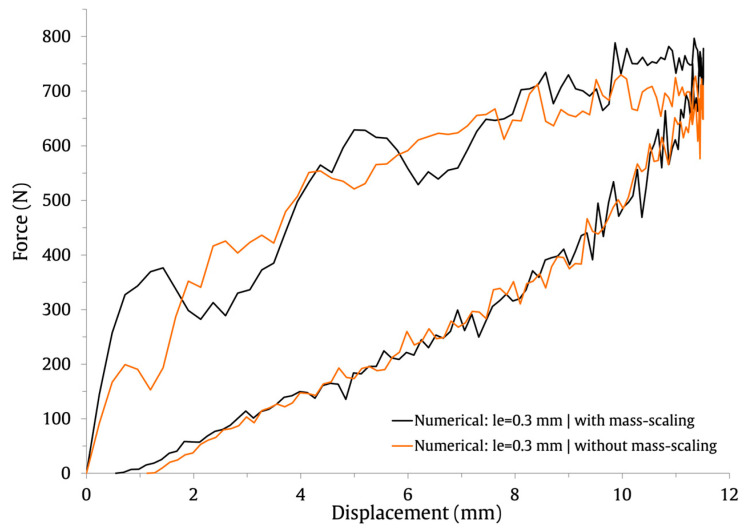
Effect of mass scaling on the force–displacement impact curves.

This baseline FE model contains approximately half a million elements and one million nodes. Therefore, to shorten the computing time for the solutions, a semi-automatic mass scaling was uniformly applied to the entire model with a target time increment of $1\times 10^{-7}$. Notice that mass scaling artificially increases the mass of the structure to reduce the frequency of the dynamic response and allows the time step of the simulation to be increased. In this study, a mass increase of 1.8% was obtained for the baseline FE model, resulting in a reduction in the computational cost of the solutions of about 51%. To assess the impact of mass scaling on the load and energy history curves, the results obtained with the baseline FE model are shown in Figure 3 (force–time), Figure 4 (energy–time) and Figure 5 (force–displacement).

It is possible to observe that these curves include oscillations brought on by the elastic wave and produced by the models’ vibrations [45,46]. The numerical predictions for the numerical maximum load, maximum displacement, and contact time are compared in Table 3.

It can be observed that the impact response of the curves is similar with and without mass scaling. Its application has a negligible effect on the contact time and no impact on the maximum displacement. Only for the maximum force do the numerical predictions differ by 8.8%; its value is higher when mass scaling is employed. Overall, the results indicate that the defined semi-automatic mass-scaling parameters have an acceptable impact on the numerical predictions and significantly lower the computational cost of the solutions.

**Figure 6:**
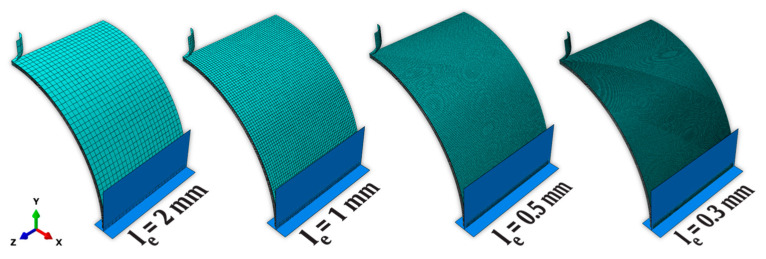
FE models used to study the effect of the FE mesh discretization.

To determine if a coarser FE mesh discretization would produce a good trade-off between the accuracy of the solution and computing cost, a parametric study was performed using increasingly coarser FE meshes, ranging from $l_{e}=0.3\;\mathrm{mm}$ to $l_{e}=2\;\mathrm{mm}$. The FE models employed to analyze the effect of the FE mesh discretization are shown in Figure 6.

**Figure 7:**
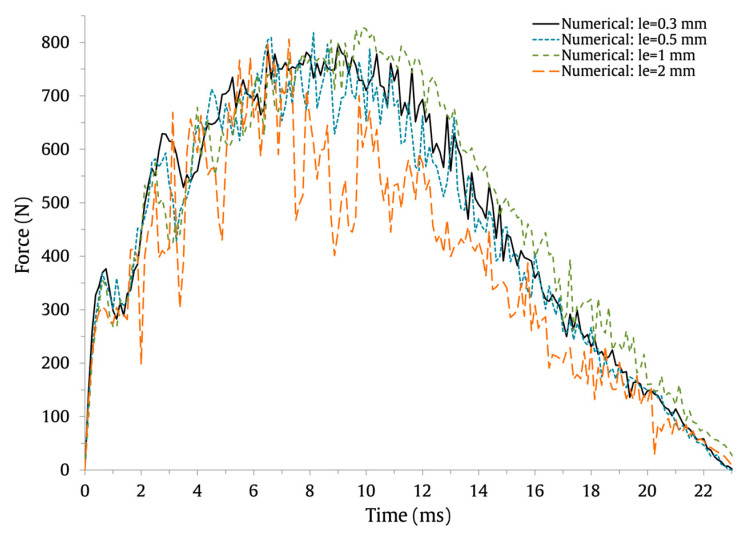
Effect of the FE mesh discretization on the low-velocity impact response of the force–time impact curves.

**Figure 8:**
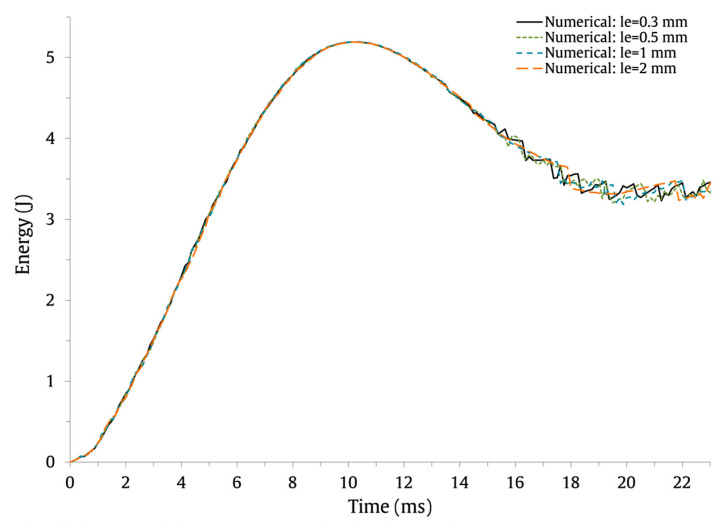
Effect of the FE mesh discretization on the low-velocity impact response of the energy–time impact curves.

**Figure 9:**
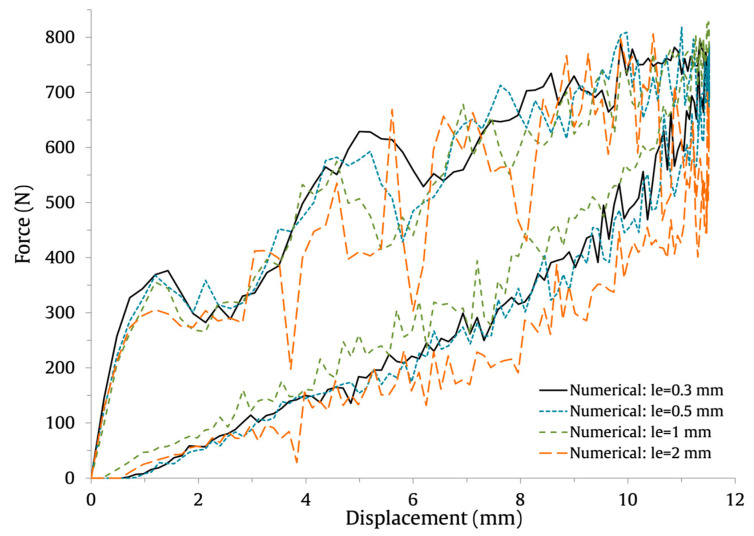
Effect of the FE mesh size on the low-velocity impact response of the force–displacement impact curves.

The numerically predicted force–time and energy–time results are shown in Figure 7 and Figure 8, respectively, and the force–displacement results are shown in Figure 9. The results show that the use of coarser FE meshes ($l_{e}=1\;\mathrm{mm}$ and $l_{e}=2\;\mathrm{mm}$) induces higher oscillations on the force–time and force–displacement curves. Nonetheless, the maximum force, maximum displacement, and contact time values are barely affected. This can be observed in Table 4, where the values and percentage difference between $l_{e}=0.3\;\mathrm{mm}$ and the remaining element lengths are presented. 

Therefore, it is clear that the FE mesh discretization, within the studied range, has a negligible impact on the load history maximum values. However, it is important to assess its effect on the interlaminar damage predictions. For this purpose, the output identifier CSQUADSCRT was used to measure the damage initiation in the cohesive surfaces. This variable indicates if the quadratic contact stress damage initiation criterion presented in Equation (9) has been satisfied. When its value reaches 1, damage in the cohesive surface is predicted to initiate. The scalar stiffness degradation for cohesive surfaces, output identifier CSDMG, was used to measure delamination after damage initiation. When it reaches the value of 1, the interface can be considered as fully delaminated (complete debonding).

**Figure 10:**
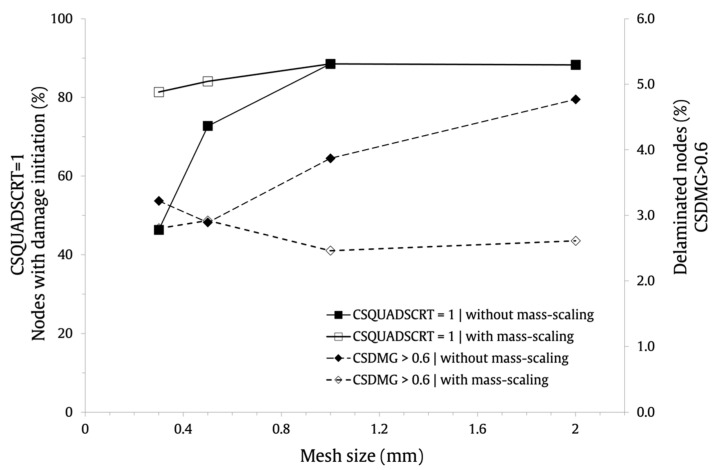
Effect of FE mesh size and mass scaling on the interlaminar damage.

The effect of the FE mesh discretization and mass scaling on the delamination initiation and progression is shown in Figure 10. The results are expressed in terms of the percentage of nodes of the 3D FE mesh for which the CSQUADSCRT is equal to 1 and for those where the CSDMG is higher than 0.6. It can be appreciated that the percentage of nodes where interlaminar damage initiation is predicted decreases for finer FE mesh discretization. If mass scaling is employed, this behavior will be especially obvious. The results indicate different behavior for the fraction of delaminated nodes with and without mass scaling. With mass scaling, the percentage of delaminated nodes slightly increases with the FE mesh refinement but reduces without it. However, for the baseline FE mesh discretization, that is, $l_{e}=0.3\;\mathrm{mm}$, the percentage of delaminated nodes is comparable: 2.8% and 3.22% with mass scaling and without mass scaling, respectively.

Data suggest that the choice of the FE mesh size does not have a significant impact on the maximum load, maximum displacement, or contact time. However, it affects the interlaminar damage initiation and progression. Consequently, it is recommended to apply the equations suggested by Turon et al. [31] to compute the FE mesh size in order to assure accurate numerical predictions of delamination. The results indicate that the defined semi-automatic mass scaling parameters have no significant impact on the numerical predictions. In addition, taking into consideration the fact that using mass scaling reduces the computational cost of the solutions by around 51%, its application is recommended.

## 6. Numerical–Experimental Correlation

The numerical results obtained with the nine-layer composite laminates FE model with mass scaling and with the different FE mesh sizes are compared with the experimental data presented in [16]. Notice that a higher post-processing by the drop-tower is responsible for the experimental curves’ higher smoothness. The numerical predictions and experimental results are summarized in Table 5 (maximum force, maximum displacement, contact time). It can be observed that the maximum load is slightly overestimated by all the numerical models, presenting errors ranging from 5% to 8.6%, among which the FE model with $l_{e}=0.3\;\mathrm{mm}$ was the closest to the experimental averaged value. The maximum displacement is not affected by the FE mesh. Its value is correctly predicted with an error of 2.7% for all FE models. This is due to the fact that the displacement is controlled by the same experimental velocity–time curve that was incorporated to the FE models. The contact time is negligibly affected by the FE mesh size. The numerical predictions are slightly overestimated, presenting an error ranging from 10.4% to 12.3%. Although all FE models present comparable values, the one that best fits the experimental evidence is obtained with $l_{e}=0.3\;\mathrm{mm}$. Moreover, taking also into consideration the results presented in Figure 10, in which it was observed that the FE mesh size considerably affects the interlaminar damage initiation and progression, the use of $l_{e}=0.3\;\mathrm{mm},$ calculated using the equations of Turon et al. [31], is recommended.

**Figure 11:**
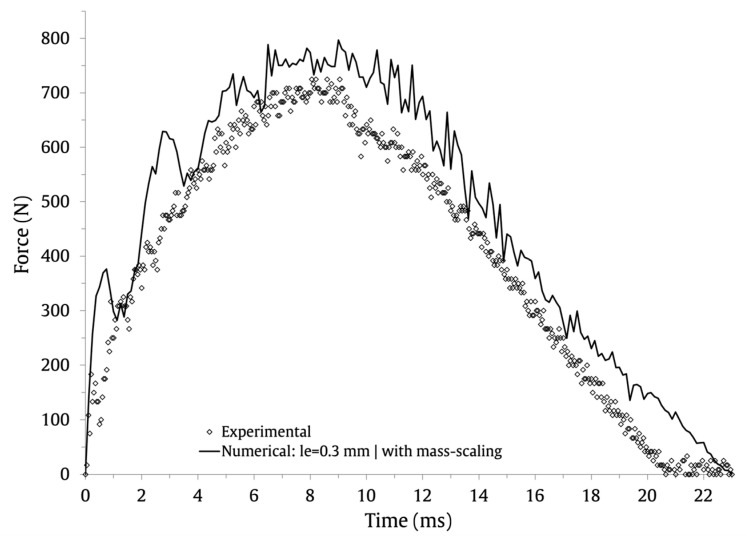
Numerical and experimental force-time results.

**Figure 12:**
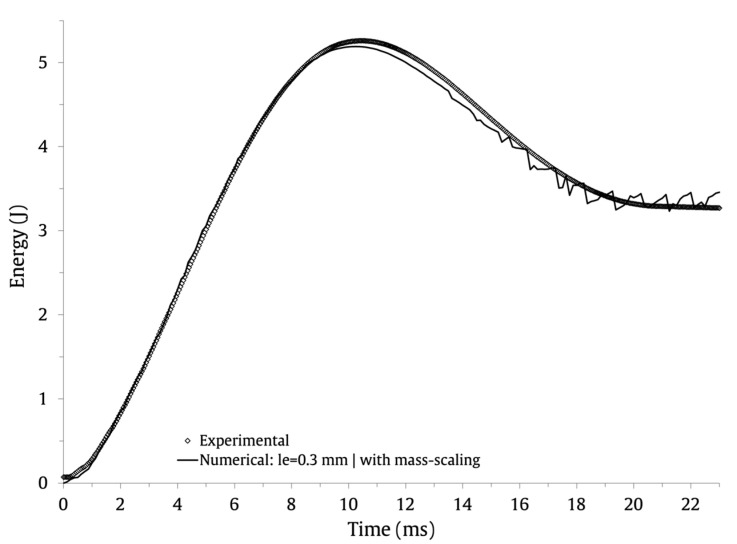
Numerical and experimental energy-time results.

**Figure 13:**
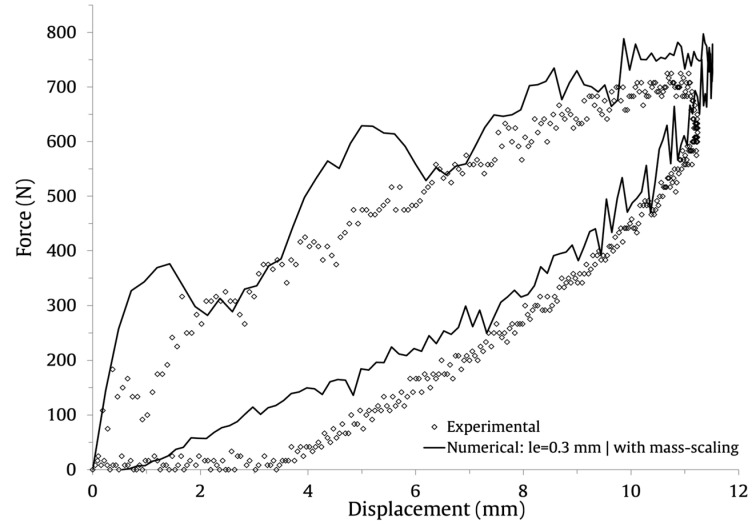
Numerical and experimental force-displacement results.

The numerical and experimental results with $l_{e}=0.3\;\mathrm{mm}$ are compared graphically in Figure 11 (force–time), Figure 12 (energy–time) and Figure 13 (force–displacement). The numerical and experimental curves, during the loading and unloading stages, show a very satisfactory agreement.

## 7. Conclusions

Finite element models were generated to study the low-velocity impact response of semicylindrical woven composite laminate shells using ABAQUS/Explicit. These FE models represent a nine-layer laminate with a thickness of 1.6 mm. A continuum damage model at the lamina level to account for intralaminar damage and a surface-based cohesive damage model at the laminas’ interface to account for interlaminar damage were used as the two constitutive models to simulate the material damage brought on by low-velocity impact load. The premise for this study was the authors’ previous work on the experimental evaluation of the effect of thickness on the impact response of semicylindrical composite laminated shells. As a result, the FE models were tested against the experimental findings for validation purposes. In this way, the stiffness properties of the woven fabric composite laminas were estimated from the constituents’ properties of the tested specimens using a homogenization process, while the reaming properties were obtained in the literature. The developed FE models require a fine FE mesh discretization to properly define the interlaminar damage behavior, making them computational expensive. Therefore, numerical simulations were carried out to find an acceptable trade-off between the accuracy of the predictions and the computational cost of the solutions. An efficient approach is employed taking advantage of the model symmetries, continuum shell elements, which have lower computing costs than solid elements, and cohesive surfaces, which do not require element definition. Moreover, the FE mesh discretization and mass-scaling technique were also examined to ascertain how they affect the effectiveness and reliability of the FE model.

The results obtained indicate that the low-velocity impact response of semicylindrical woven composite laminate shells can be reliably predicted using the aforementioned FE models. Furthermore, the mass-scaling technique is successfully used to increase the stable time increment without compromising the accuracy of the dynamic response. Its implementation reduced the computing cost of the solutions by about 51%. The maximum load, maximum displacement, and contact time do not appear to be significantly affected by the choice of FE mesh size. Yet, it has an impact on the initiation and development of interlaminar damage. In order to ensure accurate numerical predictions of delamination, it is advised to use the formula proposed by the literature to compute the characteristic length of the elements of the FE mesh.

The load and energy histories were put to the test in comparison to the experimental findings from low-velocity impacts on specimens with identical elastic properties. A satisfactory correlation between the numerical outcomes and the experimental data was observed. The results show that the maximum load and contact time are slightly overestimated by the FE model, while the maximum displacement is correctly predicted. Nevertheless, all the numerical predictions are comparable with the experimental data.

The numerical simulations complement the previous experimental work, and the developed FE models can be used for further studies, for example, to analyze the response to multi-impacts, the effect of boundary condition or how the geometric parameters of the shells, such as thickness, length, or curvature, affect the impact response of woven fabric composite laminate shells.

## Figures and Tables

**Table 1:** Intralaminar properties.

Property	Symbol	Units	Value
Density	ρ	kg/m^3^	1900
Stiffness properties	$E_{1+}=E_{1-}$	GPa	21.9
	$E_{2+}=E_{2-}$	GPa	21.9
	$E_{3}$	GPa	8.6
	$G_{12}$	GPa	3.4
	$G_{13}$	GPa	2.4
	$\nu_{12}$	-	0.14
Strength properties	$X_{1+}=X_{2+}$	MPa	250
	$X_{1-}=X_{2-}$	MPa	200
	S	MPa	40
Fracture toughness	$G_{f}^{\alpha }$	N/mm	4500
Shear plasticity	$d_{12}^{max}$	-	1
	${\tilde{\sigma }}_{y0}$	MPa	25
	C	-	800
	p	-	0.552

**Table 2:** Interlaminar properties.

Property	Symbol	Units	Value
Stiffness properties	$k_{n}=k_{s}=k_{t}$	N/mm^3^	10^6^
Strength properties	$\tau_{n}^{0}$	MPa	15
	$\tau_{s}^{0}=\tau_{t}^{0}$	MPa	30
Fracture toughness	$G_{Ic}$	N/mm	0.3
	$G_{IIc}=G_{IIIc}$	N/mm	0.6
	η	-	1.45

**Table 3:** Effect of mass scaling on the numerical predictions of maximum load, maximum displacement, and contact time.

	Max. Load (N)	Dif. ^1^ (%)	Max. Displacement (mm)	Dif. ^1^ (%)	Contact Time (ms)	Dif. ^1^ (%)
With mass scaling	797	-	11.5	-	23	-
Without mass scaling	730	8.8	11.5	0	22.6	1.8

^1^ Dif. = Difference.

**Table 4:** Effect of FE mesh discretization on the numerical predictions of maximum load, displacement, and contact time.

Mesh Size (mm)	Max. Load (N)	Difference (%)	Max. Displacement (mm)	Difference (%)	Contact Time (ms)	Difference (%)
0.3	797	-	11.5	-	23	-
0.5	818	2.6	11.5	0	23	0
1	828	3.8	11.5	0	23.5	2.2
2	806	1.1	11.5	0	23.1	0.4

**Table 5:** Numerical and experimental comparison.

Mesh Size (mm)	Maximum Load (N)			Maximum Displacement (mm)			Contact Time (ms)		
	Num.	Exp.	Error (%)	Num.	Exp.	Error (%)	Num.	Exp.	Error (%)
0.3	797	757	5.0	11.5	11.2	2.7	23	20.6	10.4
0.5	818		7.5	11.5		2.7	23		10.4
1	828		8.6	11.5		2.7	23.5		12.3
2	806		6.1	11.5		2.7	23.1		10.8

## Data Availability

Not applicable.

## Nomenclature

**Symbol**	**Description**	**Symbol**	**Description**
ρ	Density	${\tilde{\sigma }}_{0}$	Shear hardening function
$E_{1}$	Young’s modulus along fiber direction 1	$r_{\alpha }$	Axial damage thresholds
$E_{2}$	Young’s modulus along fiber direction 2	$r_{12}$	Shear damage threshold
$E_{2}$	Though-thickness Young’s modulus	$X_{\alpha }$	Tensile/compressive strength along the fiber directions
$G_{12}$	In-plane shear modulus	$d_{1}$	Tensile/compressive damage variable along direction 1
$G_{13}$	Out of plane shear modulus	$d_{2}$	Tensile/compressive damage variable along direction 2
$\nu_{12}$	In-plane Poisson’s ratio	$d_{12}$	Shear damage variable
$X_{1+}$	Tensile strength along direction 1	$g_{0}^{\alpha }$	Elastic energy density
$X_{1-}$	Compressive strength along direction 1	$L_{e}$	Characteristic length of the element
$X_{2+}$	Tensile strength along direction 2	${\bar{\varepsilon }}^{pl}$	Plastic strain due to shear deformation
$X_{2-}$	Compressive strength along direction 2	$k_{n}$	Elastic normal interlaminar stiffness
$S_{12}$	In-plane shear strength	$k_{s}$	Elastic shear interlaminar stiffness
$G_{f}^{\alpha }$	Intralaminar fracture toughness along direction 1 and 2	$k_{t}$	Elastic tangential interlaminar stiffness
$\alpha_{12}$	Parameter in the equation of shear damage	$\tau_{n}^{0}$	Maximum normal contact stress
$d_{12}^{max}$	Maximum shear damage	$\tau_{s}^{0}$	Maximum 1st shear contact stress
C	Coefficient in hardening equation	$\tau_{t}^{0}$	Maximum 2nd shear contact stress
lp	Power term in hardening equation	$G_{Ic}$	Interlaminar normal fracture toughness
$F_{\alpha }$	Axial damage activation function	$G_{IIc}$	Interlaminar 1st shear fracture toughness
$F_{12}$	Shear damage activation function	$G_{IIIc}$	Interlaminar 2nd shear fracture toughness
$F_{pl}$	Plasticity activation function	𝜂	Benzeggagh–Kenane exponent
${\tilde{\sigma }}_{\alpha }$	Effective tensile/compressive stress	μ	Friction coefficient
${\tilde{\sigma }}_{y0}$	Initial effective shear yield stress	*M*	Parameter defined for the cohesive zone model
${\tilde{\sigma }}_{12}$	Effective shear stress	$N_{e}$	Number of elements in the cohesive zone
